# Impact of Cognitive Remediation Therapy on Neurocognitive Processing in Anorexia Nervosa

**DOI:** 10.3389/fpsyt.2018.00096

**Published:** 2018-03-20

**Authors:** Jenni Leppanen, James Adamson, Kate Tchanturia

**Affiliations:** ^1^Department of Psychological Medicine, Institute of Psychiatry, Psychology, and Neuroscience, King’s College London, London, United Kingdom; ^2^South London and Maudsley NHS Foundation Trust, London, United Kingdom; ^3^Department of Psychology, Illia State University, Tbilisi, Georgia

**Keywords:** neurocognition, central coherence, executive functioning, anorexia nervosa, cognitive remediation therapy

## Abstract

**Background:**

Anorexia nervosa (AN) is characterized by severe malnutrition as well as inefficiencies in neurocognitive functioning, which are believed to contribute to the maintenance of disordered eating. The aim of this study was to examine the impact of individual cognitive remediation therapy (CRT) on neurocognition in AN.

**Methods:**

A total of 145 adult women from an eating disorders inpatient program took part in the present study. All participants were given individual CRT in addition to treatment as usual. Neurocognitive processes were assessed at baseline and at the end of treatment using task-based and self-report measures. The task-based measures included the Rey-Osterrieth Complex Figure test and the Brixton test, which were used to assess central coherence and set-shifting. The Detail and Flexibility Questionnaire was used to examine patients self-reported detail focus and cognitive flexibility.

**Results:**

Participants showed significant improvement in task-based measures of neurocognition following CRT. There were no significant changes in self-report measures.

**Conclusion:**

These findings suggest that CRT may be an effective intervention targeting inefficiencies in neurocognition in AN. Future studies may benefit from assessing neural changes associated with these improvements and conducting randomized controlled trials to replicate these findings.

## Introduction

Anorexia nervosa (AN) is a complex eating disorder, with illness progression food avoidance and malnutrition lead to functional and structural changes in the brain, such as cortical thinning and volume loss (1, 2). As a result inefficiencies in neurocognitive processing, including increased rigidity and poor global processing, begin to emerge (3–5). Indeed, a wealth of experimental work has documented poorer performance in measures of central coherence and cognitive flexibility in people with AN relative to healthy individuals (3–6). As changes in thinking styles are believed to contribute to treatment resistance in AN (1), interventions targeting neurocognitive processes are needed (7).

Cognitive remediation therapy (CRT) is an intervention that has been recently tailored for people with eating disorders to target difficulties in neurocognitive processes and encourage neurorehabilitation in AN (8). CRT is designed to be a brief intervention delivered as an adjunct treatment in addition to nutritional treatment and consists of exercises focusing on thinking strategies and processes, encouraging flexible, big picture thinking (8). Two systematic reviews have shown that CRT can be helpful in improving central coherence and set-shifting in both young and adult patients with AN (9, 10). However, it is of importance to note that the majority of the work examining the efficacy of CRT in the treatment of AN have consisted of case studies and a small case series making it difficult to draw firm, generalizable conclusions. Therefore, larger studies are needed to examine the effects of CRT on neurocognitive processing in AN.

In the present study, we aimed to examine the effect of an individual CRT intervention on neurocognitive processes in a large cohort of adult women with AN receiving inpatient treatment. We also explored the potential confounding effects of increases in body mass index (BMI) as a result of inpatient treatment in this group of women. We hypothesized that the CRT intervention would lead to significant improvement in neurocognitive functions, which cannot be explained by an increase in BMI alone.

## Methodology

### Participants

The naturalistic sample, collected over 6 years, consisted of 145 women with AN diagnosed by a consultant psychiatrist. All participants were inpatients at the South London and Maudsley NHS Foundation Trust national eating disorders service. All inpatients through the years of 2011–2017 were offered the opportunity to take part in the CRT intervention. Only those inpatients who took part in the CRT and agreed to complete the baseline and end of treatment assessments were included in the sample. Only inpatients with a primary diagnosis of AN were included. The study was approved by the National Research Ethics Service, Fulham, London (NRES-14/LO/2131). Prior to taking part, all participants gave written informed consent in accordance with the latest version of the Declaration of Helsinki. All participants engaged in individual CRT, designed to specifically target thinking styles in AN (8). The sample mean age was 25.0 (6.8) years with mean BMI of 14.9 (1.5).

### CRT Intervention

All participants were offered individual CRT to be completed alongside standard inpatient treatment, consisting of weight restoration, occupational and psychological treatment program, typically inpatient admissions last 12–14 weeks. The intervention consisted of eight or ten sessions (depending on the agreed time for the patient to stay on the ward) and was delivered by a trained psychologist over an 8-week period. Each session included six to ten exercises from the CRT manual (depending on the patients’ physical and psychological status) (8), all of which target thinking styles and strategies. At the end of each session the psychologist would encourage the patient to think about how these exercises and strategies learned through them could relate to their everyday life. As the intervention was offered as part of inpatient treatment, some participants were discharged prior to the completion of CRT, leading to a high dropout rate (60%).

### Measures

Various aspects of participants’ neurocognitive processing were assessed at baseline prior to the commencement of CRT and at the end of treatment using both task-based and self-report measures. The following neurocognitive processes were assessed using task-based measures: central coherence, as assessed with the Rey-Osterrieth Complex Figure test (5, 11, 12) and a set-shifting task as a measure of cognitive flexibility, the Brixton test (13). These measures have previously been successfully used to assess neurocognition and they have been deemed to be sensitive to detect cognitive difficulties among people with AN, as shown in two large dataset publications (3, 5).

Cognitive rigidity and attention to detail were assessed using a self-report questionnaire, the Detail and Flexibility Questionnaire [Dflex (14)]. This questionnaire has been specifically developed to assess these aspects of neurocognition among people with eating disorders (14). It has been suggested to be sensitive enough to detect changes in thinking styles after brief interventions (15).

Information regarding demographic and clinical characteristics of the sample were collected from the inpatient audit database. The following questionnaires were used to collect clinical information about eating disorder psychopathology, anxiety and depression: the Eating Disorders Examination questionnaire [EDEQ (16)] and the Hospital Anxiety and Depression Scale [HADS (17)]. Other than BMI, self-reported clinical information was only available from patients’ admission and discharge audit questionnaires.

### Statistical Analysis

All statistical analyses were conducted using R (18). Mixed effects regression was conducted to demonstrate that there was a significant increase in BMI from start to the end of intervention, which was likely due to the fact that all participants were inpatients going through weight restoration. Linear mixed effects analyses were conducted to investigate changes in central coherence, executive functioning, cognitive rigidity and attention to detail. Time (baseline, end of treatment) was entered as a fixed effect predictor and BMI was included as a covariate to explore its potential impact on neurocognitive functioning. Behavioral neurocognitive assessments were available from 145 participants at baseline and 87 participants at end of treatment. Self-report, Dflex responses were available from 49 participants at baseline and 28 participants at the end of treatment. The effect size estimated using standardized mean change and prior probability of the alternative hypothesis was also calculated for each analysis of interest as recommended by Colquhoun (19). The prior probability was calculated assuming a false positive rate of 0.05, and using the *p*-value and effect size estimate form each analysis. The prior probability indicates the probability with which we needed to approach the study to reach the present results. It can be used to give additional evidence against the null hypothesis and to support significant findings.

## Results

Participant characteristics are presented in Table 1. All participants provided their age and BMI at the start of CRT. 139 of the participants reported their duration of illness. Information regarding eating disorder psychopathology, anxiety and depression was available from 99 and 101 of the participants. Due to being inpatients, all participants entered the intervention with very low BMI and there was a significant increase in BMI by the end of the intervention [*t*(123) = 10.1, *p* < 0.001]. The effect size was large and the prior probability of the alternative hypothesis was very large, which was to be expected as the inpatient treatment consisted of nutritional rehabilitation [ES = 0.91, 95% CI (0.67, 1.15), P(AH) = 1.00].

**Table 1:** Participant characteristics.

Measure	Time	Sample size	Mean (SD)
Age	Start of CRT	145	25.0 (6.8)
Duration of illness	Start of CRT	139	9.2 (7.8)
EDEQ total	Start of CRT	99	3.8 (1.7)
HADS anxiety	Start of CRT	101	14.7 (5.1)
HADS depression	Start of CRT	101	25.7 (9.5)
BMI	Start of CRT	145	14.4 (1.3)
	End of CRT	87	15.6 (1.4)

*EDEQ, Eating Disorders Examination Questionnaire; HADS, Hospital Anxiety and Depression Scale; BMI, body mass index; CRT, cognitive remediation therapy*.

**Figure 1:**
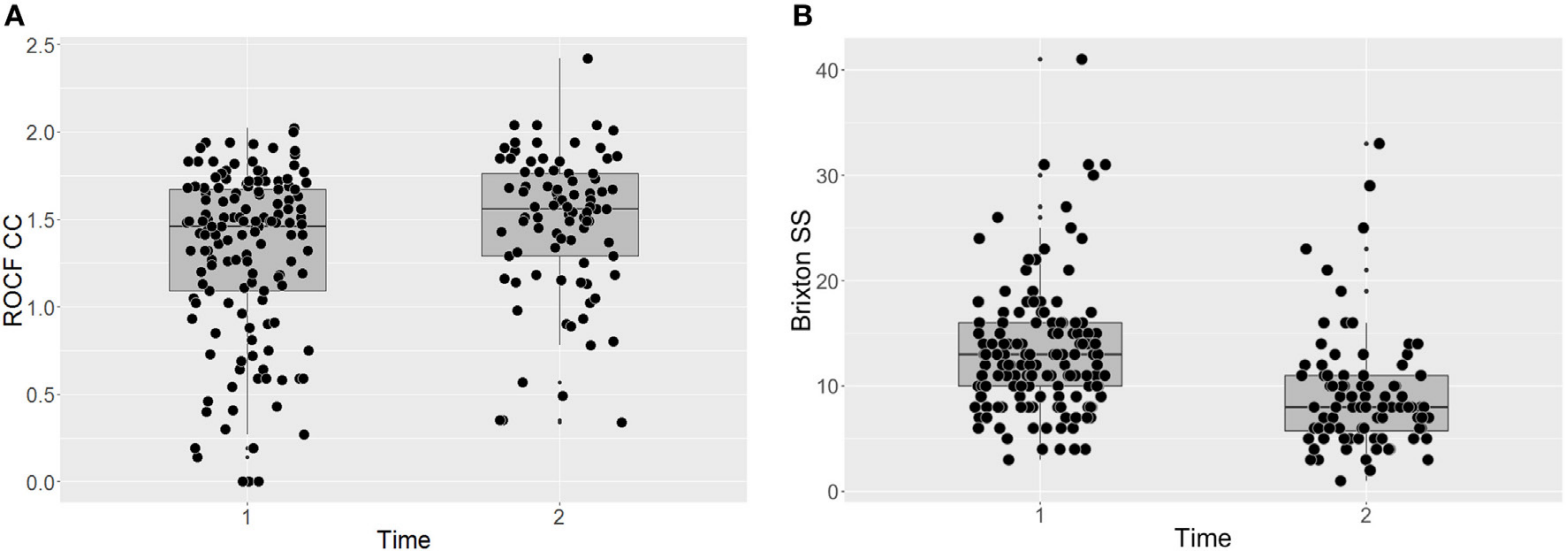
Task-based assessments of central coherence (**A**) and set-shifting (**B**) before and after CRT. ROCF, Rey-Osterrieth Complex Figure test; CC, central coherence; SS, set-shifting; Dflex, Detail and Flexibility Questionnaire; CRT, cognitive remediation therapy; Time 1, before cognitive remediation therapy; Time 2, end of cognitive remediation therapy.

Participants’ performance on the two behavioral neurocognitive tasks is presented in Figures 1A,B. The findings suggest that the individual CRT led to a significant improvement on the RCOF [*F*(140) = 10.79, p = 0.001, P(AH) = 0.17; start of treatment: *M* = 1.31 (SD = 0.48); end of treatment: *M* = 1.48 (SD = 0.41)]. The effect size was small and the prior probability suggested that we only needed to assume 17% probability that the intervention was effective to reach the present result [ES = 0.49, 95% CI (0.27, 0.72), P(AH) = 0.17]. BMI did not have a significant effect on participants’ performance on the RCOF across the time points [*F*(197) = 0.42, *p* = 0.516]. Similarly, there was a significant improvement on the Brixton test of set-shifting after individual CRT [*F*(141) = 54.79, *p* < 0.001, P(AH) = 1.00; start of treatment: *M* = 13.34 (SD = 6.01); end of treatment: *M* = 9.16 (SD = 5.54)], which was not influenced by BMI [*F*(191) = 0.60, *p* = 0.439]. The effect size was large and the prior probability was unusually high, suggesting that to reach the present results we needed to be certain that there was a significant change following the intervention, which is rarely the case [ES = 1.15, 95% CI (0.88, 1.42), P(AH) = 1.00].

**Figure 2:**
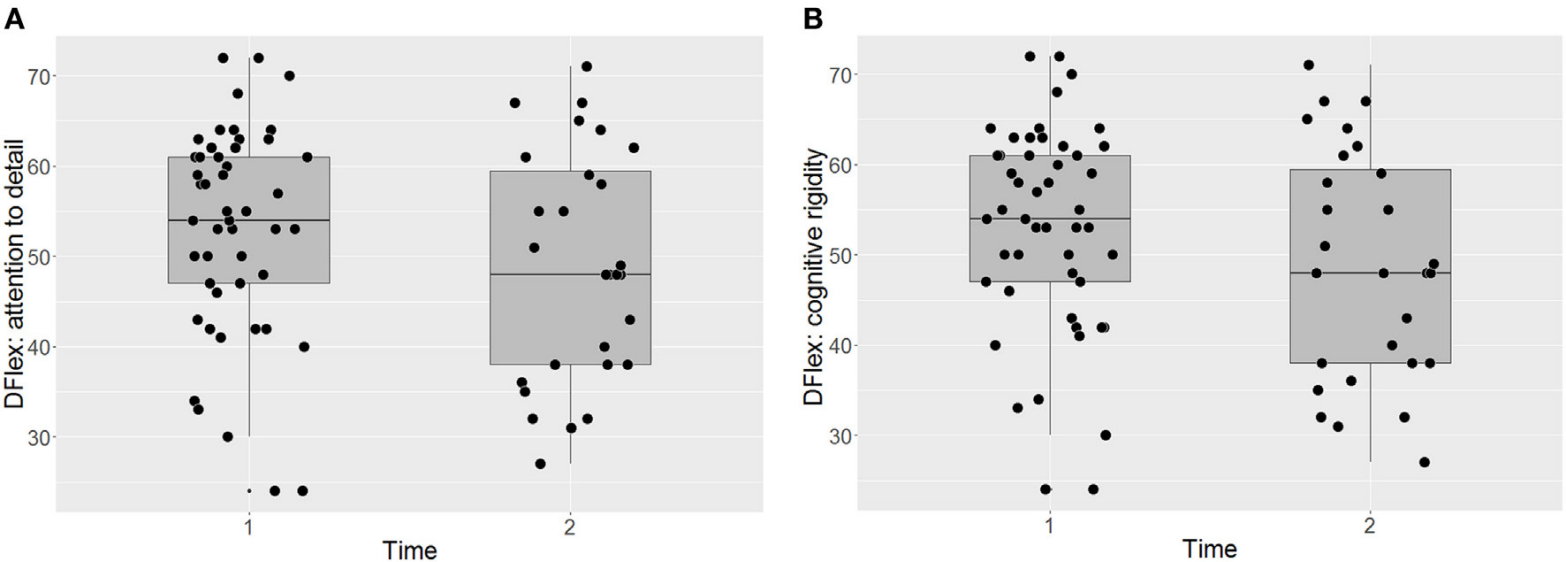
Self-report assessments of attention to detail (**A**) and cognitive flexibility (**B**) before and after CRT. Dflex, Detail and Flexibility Questionnaire; CRT, cognitive remediation therapy; Time 1, before cognitive remediation therapy; Time 2, end of cognitive remediation therapy.

Participants’ responses on the Dflex are presented in Figures 2A,B. The intervention did not have a significant impact on self-reported cognitive rigidity [*F*(38) = 1.20, *p* = 0.281 P(AH) = 0.43; start of treatment: *M* = 52.96 (SD = 11.59); end of treatment: *M* = 48.79 (SD = 12.97)] and there was no significant impact of BMI on the self-report measure across time points [*F*(61) = 0.39, *p* = 0.536]. The effect size and the prior probability of the alternative hypothesis were both small [ES = 0.45, 95% CI (0.06, 0.84), P(AH) = 0.27]. Similarly, there was no significant improvement in self-reported attention to detail [*F*(37) = 0.07, *p* = 0.793, P(AH) = 0.97; start of treatment: *M* = 46.73 (SD = 12.30); end of treatment: *M* = 45.54 (SD = 13.03)], with no significant impact of BMI [*F*(63) = 0.47, *p* = 0.494]. The effect size was negligible and the prior probability of the alternative hypothesis was very large [ES = 0.11, 95% CI (−0.26, 0.49), P(AH) = 0.97].

## Discussion

The present naturalistic study investigated the impact of individual CRT on task-based measures of central coherence and set-shifting among inpatients with AN. The CRT intervention led to a significant improvement in these neurocognitive processes, which could not be explained by an increase in BMI alone. The present findings suggest that CRT may be an effective intervention to target executive functioning and difficulties in central coherence in AN. This fits with the findings from a previous systematic review that synthesized findings from eleven case series and four randomized controlled trials (RCTs) investigating the effects of CRT in AN (9, 10). Although the case series generally had small sample sizes, the findings were encouraging showing significant improvements in a number of neurocognitive processes, including central coherence and executive functioning (9, 10). Moreover, two out of the three RCTs that investigated changes in neurocognition following CRT also reported significant improvements in people with AN (9, 10).

Similarly, CRT has been found to lead to significant improvement in neurocognition in other psychiatric disorders and brain injury. RCTs have reported significant improvements in executive functioning and central coherences following CRT when compared to control treatment in schizophrenia and depression (20, 21). Furthermore, a few studies have also reported changes in brain function in regions including the prefrontal cortex and structural improvements, such as increased gray matter volume, following CRT in schizophrenia, stroke, and traumatic brain injury (21–23). Similarly, a recent pilot studies in AN have reported changes in prefrontal cortical activation during cognitive performance following CRT (24, 25). Together, these findings suggest that CRT could be beneficial in treatment of number of psychiatric disorders and neurological conditions. However, additional RCTs investigating the mechanism underlying the effects of CRT are still needed.

Although the present study found significant improvements on task-based measures of neurocognition following CRT, based on the present findings it is difficult to determine whether CRT can be effective in supporting neurorehabilitation in AN. Two pilot studies have thus far explored the impact of CRT on brain function during central coherence (25) and set-shifting tasks (24). The studies reported somewhat contradictory findings, with one documenting significant reduction in activation in regions associated with visuospatial thinking, such as the precuneus, and in medial prefrontal regions during the central coherence task (25). The other study, did not find significant changes in brain activation following CRT (24). Although the findings appear encouraging, especially for improvements in central coherence, it is of importance to note that both pilot studies had small sample sizes. Larger neuroimaging studies are needed before firm conclusions regarding brain regions affected by CRT can be reached.

Interestingly, the present study did not find significant change in self-report measures of neurocognitive processes on the Dflex. Although this may have been due to the small sample size in this measure, this could indicate that the effects of individual CRT may be subtler and more difficult to detect with self-report measures. Furthermore, it has been documented that self-report and task-based measures of neurocognitive processes show poor correlation, suggesting that they may not be measuring the constructs in the same manner (26, 27). Future studies may benefit from exploring the different processes that self-report and task-based measures may be tapping into in order to identify the most useful measures to assess effectiveness of an intervention in RCTs.

The main limitations of this study were a lack of a control group and a small sample in the Dflex assessments. We assessed and audited CRT in routine clinical practice where some patients were not able to complete the full package of CRT or time 2 assessment leading to a higher drop-out rate than reported in the previous literature. Importantly, the prior probability of the alternative hypothesis in this was 100%, which is unusually high, possibly suggesting that there were other factors present. To validate the present findings and examine the potential impact of confounders, such as learning effects, or regression toward the mean, RCTs are needed. Additionally, future studies would also benefit from exploring whether CRT can support functional outcomes after the treatment. Another limitation of the present study was that the sample consisted of exclusively inpatients with AN and further work is needed to replicate these findings in outpatient and day care settings. Finally, we were unable to establish the types of medication the participants were taking during the intervention, which could have impacted on the results. Future studies may benefit from exploring the role of psychotropic medication on the effectiveness of CRT on neurocognition.

## Conclusion

The present naturalistic study examined the impact of a CRT intervention on neurocognition among inpatients with AN. At the start and the end of intervention, central coherence and set-shifting were assessed using task-based measures, while a self-report questionnaire was used to examine detail focus and cognitive flexibility. The findings demonstrate that there was a significant improvement in both task-based measures of neurocognition following CRT. These findings suggest that CRT may be an effective intervention targeting cognitive difficulties in AN and could be used to supplement treatment as usual. However, further research, especially large RCTs, are still needed before firm conclusions about effectiveness and the underlying mechanisms can be drawn.

## Ethics Statement

The study was approved by the National Research Ethics Service, Fulham, London (NRES-14/LO/2131). Prior to taking part, all participants gave written informed consent in accordance with the latest version of the Declaration of Helsinki.

## Author Contributions

Data analysis, wrote article: JL. Data collection, delivering intervention, and contributed to writing article: KT. Data collection, database maintenance, and contributed to writing article: JA.

## Conflict of Interest Statement

The authors declare that the research was conducted in the absence of any commercial or financial relationships that could be construed as a potential conflict of interest.
